# Functional Outcome of Lower Limb Long Bone Trauma Management in Pregnant Mothers: A Prospective Study of 30 Cases From a Tertiary Care Centre in North India

**DOI:** 10.7759/cureus.54794

**Published:** 2024-02-23

**Authors:** Dharm Pal, Nitesh Kumar, Ashim Sharma, Kuldip Sandhu, Ajay Sharma, Dharminder Singh

**Affiliations:** 1 Orthopaedics, Government Medical College, Patiala, IND; 2 Anaesthesia, Homi Bhabha Cancer Hospital, Sangrur, IND

**Keywords:** surgical intervention, prospective study, musculoskeletal injuries, trauma during pregnancy, obstetric and orthopedic trauma, orthopedic trauma, lower limb fractures

## Abstract

Introduction: The occurrence of orthopedic injuries during pregnancy carries considerable morbidity and mortality for both the mother and fetus. Successful care of lower limb fractures during pregnancy requires a multidisciplinary approach. Both operative and non-operative treatments must be taken into account by the treating orthopedic physician. There is limited literature available on the management of these lower limb fractures in pregnancy, and peri-operative management of this obstetric and orthopedic trauma is largely unclear. Trauma during pregnancy is a common cause of non-obstetrical maternal death, having a significant public health burden to both the mother and child. The aims and objectives of this study were to review the common causes of lower limb long bone trauma during pregnancy and their functional outcome in terms of morbidity and mortality. This study evaluates various operative and conservative methods of treatment to provide a comprehensive management approach to pregnant patients with lower limb trauma.

Materials and methods: A prospective study on functional outcomes of 30 pregnant females who were admitted with lower limb long bone fractures from 2017 to 2021 was done. The patients were randomly selected intra-operatively for various procedures based on the surgeon's preference. All patients were followed for two years or till union occurred, and the radiographic union score for tibial (RUST) and modified radiographic union score for tibial (mRUST) fracture criteria were used to assess bony union clinico-radiologically.

Results: During this study, the mean age of patients was 27 years (range 19-38), having right-side (53.33%) predominance with road traffic accidents (n=22) and falls (n=6) as the most common causes of injury. Two cases of domestic violence were also reported. In our study, the maximum number of cases was 17-25 weeks of their gestation; 12 (40%) patients had tibial fractures, and 18 (60%) had femoral fractures. Six tibial fractures were handled conservatively, while all femoral fractures required surgical intervention. Out of 18 femoral fractures, which were treated surgically, dynamic compression plating was done in 15 (83.33%) patients, while interlock nailing was done in three patients. Six tibial fractures have been operated upon, two (66.66%) with dynamic compression plating and four (33.33%) with an interlocking nail.

Conclusion: A multidisciplinary approach in terms of both operative and non-operative methods must be taken into account for treating pregnant mothers by the orthopedic physician while carefully weighing the benefits and risks of both procedures. Based on the pattern and displacement of the fracture, many prenatal fractures can be treated conservatively. Another alternative that is frequently safe is to postpone the surgical procedure until childbirth. The physiologic changes associated with pregnancy and any potential dangers to the fetus must be taken into account by the orthopedic surgeon when fractures necessitate surgical intervention. The surgeon is responsible for the patient's correct placement, the C-arm's use, the radiation dose, and the intra-operative fetal monitoring, as well as the danger brought on by anesthetics, antibiotics, analgesics, and anticoagulants.

## Introduction

Orthopedic injury during pregnancy is typically associated with high maternal and fetal morbidity and mortality [1,2]. It is uncommon and poorly covered in the literature to address bone fractures in pregnant patients. Orthopedic surgeons struggle with managing lower limb injuries that occur during pregnancy. It is difficult because the treating surgeon must consider both the mother and the fetus. The mother and fetus are linked; therefore, any therapy will have an impact on both of them. Pregnancy-related trauma raises non-obstetric maternal mortality [3]. Pregnant trauma patients are frequently thought to require extensive management in accordance with physiological changes occurring in their bodies. The management is made more difficult by the mother's unique anatomical and physiological changes during pregnancy [4,5]. Motor vehicle accidents (MVA), falls, assaults, and interpersonal violence are the most frequent causes [6]. It is important to keep in mind that any treatment for a patient who is pregnant and has experienced trauma will have an impact on both the mother and the fetus. Orthopedic surgeons, obstetric specialists, anesthesiologists, general trauma surgeons, the emergency medical team, and nursing personnel must all work together to undertake a multidisciplinary approach [7]. First-line management is focused on the mother. The risks of premature labor, placental abruption, miscarriage, preterm rupture of membranes, and fetal death must be considered while designing a treatment strategy for a pregnant patient with orthopedic injuries. Pregnancy-related physiological and anatomical changes must be taken into account. All of these changes affect fetal and maternal outcomes. Based on the pattern and displacement of the fracture, many prenatal fractures can be treated conservatively. Another alternative that is frequently safe is to postpone the surgical procedure until childbirth. The physiologic changes associated with pregnancy and any potential dangers to the fetus must be taken into account by the orthopedic surgeon when fractures necessitate surgical intervention. The surgeon is responsible for the patient's correct placement, the C-arm's use, the radiation dose, and the intra-operative fetal monitoring. The primary objective of the study is to evaluate the characteristics of fractures during pregnancy, while the secondary objective is to study the radiographic union score in tibial fractures using the radiographic union score for tibial (RUST) and modified radiographic union score for tibial (mRUST) fracture scoring criteria.

## Materials and methods

This study was conducted after the approval of the Institutional Ethical Committee and Scientific Committee of the Government Medical College (approval number: EC/NEW/INST/2017/997/25432). In this study, all 30 patients were included, who were admitted with lower limb long bone trauma during pregnancy in the Department of Orthopaedics, Government Medical College in Patiala, India, from January 2017 to July 2021. All patients gave informed consent before inclusion into the study and prior to the management of these cases. Pre-operative evaluation of patients in terms of age, sex, pre-morbidity status, mode of injury and fracture classification, and any other comorbidities was done. The patients were randomly selected intra-operatively for various procedures based on the surgeon's preference. Patients were operated on a fracture table with a closed or mini-open reduction technique to create a biomechanically stable osteosynthesis while using various implants. All femoral fractures were reduced on longitudinal traction, and open reduction and fixation were done in a supine or lateral position under epidural anesthesia. Some of the simple femoral fractures were managed with closed interlock nailing. All tibial fractures were reduced under C-arm and managed conservatively with plaster application or interlock nailing, which failed on closed reduction or were unmanageable conservatively. Intra-operatively, the risks of premature labor, placental abruption, miscarriage, preterm rupture of membranes, and fetal death must be considered while designing a treatment strategy for a pregnant patient with orthopedic injuries. Pregnancy-related physiological and anatomical changes must be taken into account. The duration of surgery, blood loss, and radiation dose must be considered intra-operatively. All patients were followed for two years or till union occurred, and functional outcome was measured clinico-radiologically post-operatively. Patients were assessed in terms of bony union, mobility status, shortening of limb, and its various post-operative complications. RUST and mRUST scores were used to assess the fracture union in these patients. Post-operatively, patients were evaluated clinico-radiologically on a monthly basis for six months and three months till a follow-up of two years. Microsoft Excel was used to evaluate the results, and a p-value of 0.005 was considered significant (Table 1).

**Table 1:** RUST and mRUST scoring criteria per cortex visible score is given in two orthogonal planes (antero-posterior and lateral), and the final score is the sum of four cortices. RUST: radiographic union score for tibial fractures; mRUST: modified radiographic union score for tibial fractures

	Callus	Fracture line	Score
RUST	Absent	Visible	1
	Present	Visible	2
	Present	Invisible	3
mRUST	Absent	Visible	1
	Present	Visible	2
	Bridging	Visible	3
	Remodeled	Invisible	4

Inclusion criteria

The inclusion criteria encompass pregnant females with fractures involving the lower limbs and long bones. Participants meeting this criterion had fractures with a duration of less than two weeks. The patients included in the study were ambulatory before sustaining the fracture.

Exclusion criteria

Exclusion criteria consisted of patients with pathological fractures and open fractures and those with known disorders of bone metabolism, except for osteoporosis. Any individuals with associated infectious conditions of bone were also excluded from the study. Fractures with a duration exceeding two weeks were part of the exclusion criteria. Furthermore, patients who refused to provide consent were not included in the study.

Statistical analysis

The data was entered on a Microsoft Excel sheet and evaluated using IBM SPSS Statistics for Windows, V. 21.0 (IBM Corp., Armonk, NY). All parametric data was evaluated using Student's t-test, and chi-squared/Fischer's test was used for non-parametric data. A p-value of <0.05 was taken as statistically significant. 

## Results

During this study, a total of 30 cases of lower limb long bone trauma during pregnancy were managed conservatively or surgically from January 2017 to July 2021 in the Department of Orthopaedics, Government Medical College in Patiala. From the final analysis, all patients were females having a mean age of 27 years (range 19-38) with a slight predominance in urban females. Road traffic accidents were the most common mode of injury (n=22), followed by simple falls (n=6). Two cases of domestic violence were also reported in this study. All cases were of long bone shaft fractures (Table 2).

**Table 2:** Various modes of injury. RTA: road traffic accidents

Mode of injury	Number of cases
Falls	6
RTA	22
Domestic violence	2
Total	30

Table 3 categorizes the cases into two sides, "LEFT" and "RIGHT," with 14 cases affecting the left side and 16 cases affecting the right side. The "Total" row at the bottom indicates that there are 30 cases in total.

**Table 3:** Sides of the limb affected.

Side	Number of cases
LEFT	14
RIGHT	16
Total	30

All cases were of long bone shaft fractures. The femur was the most common long bone fracture (n=18) compared to tibial fractures (n=12). During this study, all femoral fractures were treated surgically, while half of the tibial fractures (n=6) were managed conservatively (Table 4). 

**Table 4:** Types of lower limb trauma.

Type	Number of cases
Femoral fractures	18
Tibial fractures	12
Total	30

Table 5 summarizes the number of cases in different gestational periods. It shows that there were no cases of gestational periods less than eight weeks or beyond 40 weeks. Most cases occurred between 17 and 34 weeks, with the highest count at 17-25 weeks (14 cases) and the second highest at 26-34 weeks (11 cases). There were two cases between nine and 16 weeks and three cases between 35 and 40 weeks. 

**Table 5:** Gestation in weeks.

Gestation in weeks	Number of cases
<8	0
9-16	2
17-25	14
26-34	11
35-40	3
41-44	0

Table 6 displays the type of interventions performed on pregnant females for femoral and tibial fractures. All femoral fractures were treated operatively, while tibial fractures were split equally between operative and non-operative methods. 

**Table 6:** Type of intervention done in pregnant females.

Type of intervention	Femoral fractures		Tibial fractures	
	Number	%age	Number	%age
Operative	18	100	6	50
Non-operative	0	0	6	50
Total	18	100	12	100

RUST and mRUST scores were used to evaluate periodically and found satisfactory in a given time for long bone fractures. RUST and mRUST criteria have been used to assess fracture union. A minimum score of 4 and a maximum of 12 are there. The prerequisite for union is to provide a favorable biomechanical environment to unite. This score depends on various factors like age, sex, soft tissue damage, displacement and comminution, nutritional deficiencies, or any other endocrinopathies. RUST score is a novel assessment tool to standardize the radiographic union of long bone (Table 7).

**Table 7:** Number of cases showing RUST and mRUST scoring criteria. RUST: radiographic union score for tibial fractures; mRUST: modified radiographic union score for tibial fractures

RUST/mRUST score per cortex	Number of patients at six months	Number of patients at one year	Number of patients at two years
4	14	2	-
8	12	6	-
12	4	22	30

## Discussion

Orthopedic injury during pregnancy is a rare occurrence, with a 1-6% frequency [8,9]. Despite being rare, it is linked to high rates of maternal and fetal morbidity and mortality. Given the intricacy of the pregnant lady, orthopedic trauma provides difficulty for the orthopedic surgeon. First-line management is focused on the mother. It is important to keep in mind that any treatment for a patient who is pregnant and has experienced trauma will have an impact on both the mother and the fetus. Orthopedic surgeons, obstetric specialists, anesthesiologists, general trauma surgeons, the emergency medical team, and nursing personnel must all work together to undertake a multidisciplinary approach [10].

A multidisciplinary team must include an obstetrician expert for the initial evaluation, stabilization, and ongoing management of a pregnant trauma patient. Our study focuses on the treatment of lower limb long bone trauma in pregnant women, and we employ a multidisciplinary approach to patient management. The need for additional intervention, which may be surgical or conservative, is determined following the initial stabilization of the pregnant patient with lower limb damage. Additionally, the proper anesthetic to be utilized was decided. Neuroaxial anesthesia is mostly used [11]. 

The management of pregnant patients with lower limb injuries relies heavily on intra-operative care and surveillance. The patient's correct placement, the C-arm's use, the radiation dose, and the intra-operative fetal monitoring are crucial treatment procedures. Because it prevents inferior vena cava compression by a gravid uterus, the left lateral position is thought to be safer [12]. With the help of neonatologists and obstetricians, the multidisciplinary approach also takes into account continuous intra-operative fetal monitoring [13].

During surgery, the maternal abdomen is shielded from dangerous radiations using a lead apron. The implant type also influences radiation exposure. Less radiation is needed to treat fractures with plates as opposed to intra-medullary nails [14]. Only three intra-medullary nails were used for femoral fractures among the 18 patients, while plating was used for the remaining 15 patients. Based on the fracture pattern and displacement, a plaster cast can be used to address tibial fractures. Six of the 12 tibial fractures require surgery. Out of the six instances that were operated on, two had plating, and four had an interlocking nail in the tibia. All procedures had included very little radiation exposure [15,16].

All femoral and nine tibial patients had shown union clinico-radiologically in an appropriate time on follow-up of two years. Out of three tibial fractures that were treated by interlock nails, two had shown delayed union, which was managed with the dynamization of the implant. RUST and mRUST criteria have been used to assess fracture union. These depend on various factors like age, sex, soft tissue damage, displacement and comminution, nutritional deficiencies, or any other endocrinopathies. RUST score is a novel assessment tool to standardize the radiographic union of long bones.

## Conclusions

Our study's findings suggest that a multidisciplinary approach is necessary for the effective treatment of lower limb fractures during pregnancy. Both operative and non-operative treatments must be taken into account by the treating orthopedic physician. Carefully weighing the benefits and risks of surgical intervention is necessary. Based on the pattern and displacement of the fracture, many prenatal fractures can be treated conservatively. Another alternative that is frequently safe is to postpone the surgical procedure until after birth.

The physiologic changes associated with pregnancy and any potential dangers to the fetus must be taken into account by the orthopedic surgeon when fractures necessitate surgical intervention. The surgeon is responsible for the patient's correct placement, the C-arm's use, the radiation dose, the intra-operative fetal monitoring, and, additionally, the danger brought on by anesthetics, antibiotics, analgesics, and anticoagulants. Due to its ability to reduce fetal hypotension, the left lateral decubitus posture is thought to be safer. Overall, we can state that a multidisciplinary team, including orthopedic surgeons, obstetric specialists, anesthesiologists, general trauma surgeons, the emergency medical team, and nursing staff, must be deployed.
